# Performance of spirometry assessment at TB diagnosis

**DOI:** 10.5588/ijtld.23.0040

**Published:** 2023-11-01

**Authors:** A. Rachow, O. Ivanova, A. Bakuli, C. Khosa, P. Nhassengo, O. Owolabi, S. Jayasooriya, N. E. Ntinginya, I. Sabi, M. Rassool, J. Bennet, S. Niemann, A-M. Mekota, B. W. Allwood, R. S. Wallis, S. Charalambous, M. Hoelscher, G. Churchyard

**Affiliations:** 1 Division of Infectious Diseases and Tropical Medicine, Medical Centre of the University of Munich, Munich; 2 German Center for Infection Research (DZIF), Partner Site Munich, Munich, Germany; 3 Instituto Nacional de Saúde, Marracuene, Mozambique; 4 Vaccines and Immunity Theme, Medical Research Council Unit The Gambia at the London School of Hygiene & Tropical Medicine, Fajara, The Gambia; 5 Mbeya Medical Research Centre, National Institute for Medical Research, Mbeya, Tanzania; 6 Clinical HIV Research Unit, Department of Internal Medicine, School of Clinical Medicine, Faculty of Health Sciences, University of Witwatersrand, Johannesburg, South Africa; 7 Leibniz Lung Center, Research Center Borstel, Borstel, Germany; 8 Division of Pulmonology, Department of Medicine Stellenbosch University and Tygerberg Hospital, Cape Town; 9 The Aurum Institute, Johannesburg; 10 Department of Medicine, Vanderbilt University, Nashville, TN, USA

**Keywords:** spirometry, tuberculosis, lung impairment, post-TB lung disease, low- and middle-income countries

## Abstract

**BACKGROUND:**

Spirometry is considered relevant for the diagnosis and monitoring of post-TB lung disease. However, spirometry is rarely done in newly diagnosed TB patients.

**METHODS:**

Newly diagnosed, microbiologically confirmed TB patients were recruited for the study. Spirometry was performed within 21 days of TB treatment initiation according to American Thoracic Society/European Respiratory Society guidelines. Spirometry analysis was done using Global Lung Initiative equations for standardisation.

**RESULTS:**

Of 1,430 eligible study participants, 24.7% (353/1,430) had no spirometry performed mainly due to contraindications and 23.0% (329/1,430) had invalid results; 52.3% (748/1,430) of participants had a valid result, 82.8% (619/748) of whom had abnormal spirometry. Of participants with abnormal spirometry, 70% (436/619) had low forced vital capacity (FVC), 6.1% (38/619) had a low ratio of forced expiratory volume in 1 sec (FEV_1_) to FVC, and 19.1% (118/619) had low FVC, as well as low FEV_1_/FVC ratio. Among those with abnormal spirometry, 26.3% (163/619) had severe lung impairment.

**CONCLUSIONS:**

In this population, a high proportion of not performed and invalid spirometry assessments was observed; this was addressed by removing tachycardia as a (relative) contraindication from the study guidance and retraining. The high proportion of patients with severe pulmonary impairment at the time of TB diagnosis suggests a huge morbidity burden and calls for further longitudinal studies on the relevance of spirometry in predicting chronic lung impairment after TB.

## Abstract (fr)

**CONTEXTE:**

La spirométrie est considérée comme pertinente pour le diagnostic et le suivi de la maladie pulmonaire post-TB. Cependant, la spirométrie est rarement pratiquée chez les patients dont la TB vient d’être diagnostiquée.

**MÉTHODES:**

Des patients nouvellement diagnostiqués, dont la TB a été confirmée microbiologiquement, ont été recrutés pour l’étude. La spirométrie a été réalisée dans les 21 jours suivant le début du traitement de la TB, conformément aux directives de l’American Thoracic Society/European Respiratory Society. L’analyse de la spirométrie a été effectuée en utilisant les équations de la Global Lung Initiative pour la standardisation.

**RÉSULTATS:**

Sur les 1 430 participants éligibles à l’étude, 24,7% (353/1 430) n’ont pas eu de spirométrie effectuée, principalement en raison de contre-indications, et 23,0% (329/1 430) ont eu des résultats non valides**;** 52,3% (748/1 430) des participants ont eu un résultat valide, 82,8% (619/748) d’entre eux ayant une spirométrie anormale. Parmi les participants présentant une spirométrie anormale, 70% (436/619) avaient une faible capacité vitale forcée (FVC), 6,1% (38/619) avaient un faible rapport entre le volume expiratoire forcé en 1 seconde (FEV_1_) et la FVC, et 19,1% (118/619) avaient une faible FVC, ainsi qu’un faible rapport FEV_1_/FVC. Parmi ceux qui présentaient une spirométrie anormale, 26,3% (163/619) avaient une déficience pulmonaire sévère.

**CONCLUSIONS:**

Dans cette population, on a observé une proportion élevée d’évaluations spirométriques non effectuées et non valides ; ce problème a été résolu en supprimant la tachycardie en tant que contre-indication (relative) des directives de l’étude et en procédant à une nouvelle formation. La proportion élevée de patients présentant une atteinte pulmonaire sévère au moment du diagnostic de la TB suggère une charge de morbidité énorme et appelle à d’autres études longitudinales sur la pertinence de la spirométrie dans la prédiction de l’atteinte pulmonaire chronique après la TB.

While significant effort has been made worldwide by national TB programmes (NTPs) to improve the diagnosis and treatment of TB, less attention has been paid to post-TB lung disease (PTLD).^1^,^2^ No evidence-based guidelines addressing PTLD are currently available. However, given the significant morbidity and mortality associated with PTLD,^3^,^4^ evidence is required on the prevention and management, both during and after TB treatment.^5^ In two recently published clinical standards, spirometry is considered essential for the evaluation of PTLD at the end of TB treatment.^6^,^7^ A recent meta-analysis showed abnormally impaired values for the spirometry parameters – forced vital capacity (FVC) and forced expiratory volume within 1 sec (FEV_1_) – in the majority of persons after end of TB treatment.^8^ However, to assess the development of PTLD and evaluate adjunct host-directed therapies (HDTs), spirometry data during the early phases of TB treatment are needed. Despite being the most commonly used pulmonary function test worldwide, spirometry is rarely performed in acutely diseased TB patients, as clinicians and researchers may question whether it is safe, practical and tolerable in these patients. Furthermore, spirometry is often unavailable in low- and middle-income countries. The TB Sequel project includes a prospective multi-centre African clinical cohort study assessing the development, characteristics and risk factors of PTLD in newly diagnosed TB patients.^9^ Given the challenges to conducting spirometry in ill patients in resource-constrained settings, we describe the feasibility and performance of spirometry in our study participants. We report the findings of spirometry testing for subjects with a valid result at the time of TB diagnosis.

## METHODS

Comprehensive details on recruitment and study procedures are described in the TB Sequel study protocol.^8^ Briefly, from 29 August 2017 until 27 December 2019, newly diagnosed and microbiologically confirmed TB patients were recruited within 7 days of TB treatment initiation at four research sites: the Clinical HIV Research Unit (CHRU) at the Helen Joseph Hospital in Johannesburg, South Africa; the TB research clinic of the National Institute of Medical Research, Mbeya Medical Research Centre in Mbeya, Tanzania; the Mavalane Health Centre and the Machava General Hospital of the Instituto Nacional de Saúde in Maputo, Mozambique; and the Medical Research Council Unit in Banjul, The Gambia. Baseline clinical data from all participants was collected on study visits within 14 days after enrolment. Data collection included questionnaires (demographics, medical history, including TB symptoms, and socio-economic data), clinical examination, chest X-ray (CXR), spirometry assessment and HIV/CD4 testing.

Spirometry was performed according to American Thoracic Society/European Respiratory Society (ATS/ERS) guidelines and under strict infection control measures^10^ (refer to online Supplementary Data for further information) using the ndd EasyOne^®^ (ndd Medical Technologies, Zurich, Switzerland) or Easy on-PC (ndd Medical Technologies) device. Prior to study start, dedicated clinical staff (clinicians, nurses and medical officers) were centrally trained on the principles, conduct and review of the spirometry procedure and equipment maintenance during a one-week, face-to-face, hands-on training, conducted by a specialised training expert of the Pan African Thoracic Society (PATS). On study initiation, a one-day refresher training on spirometry was performed at each study site. Remote and in-person retraining courses were conducted during the study period according to local requirements. A detailed description of the performance of spirometry, quality control procedures and statistical analysis of spirometry results, including definitions of valid tests, and the type and severity grading of lung impairment, is provided in the online Supplementary Data. Briefly, all spirometry curves were reviewed by the responsible site investigators and the external PATS expert. Only spirometry data meeting the criteria for acceptability and usability for FEV_1_ and FVC according to ATS/ERS guidelines^10^–^12^ were considered ‘valid’ and included in the further analysis. For interpretation of spirometry data, Global Lung Initiative (GLI) reference equations and the ‘others’ category were used for standardisation.^13^ Criteria for defining ventilation patterns were as follows: obstructive disease: FEV_1_/FVC ratio <lower limit of normal (LLN); restrictive disease: FEV_1_/FVC ratio ≥LLN and FVC <LLN; mixed pattern: FEV_1_/FVC ratio <LLN and FVC < LLN; normal: FEV_1_/FVC ratio ≥ LLN and FVC ≥ LLN. In participants with a relative contraindication to spirometry such as tachycardia (>100 heart beats per min) acute cardiovascular events, specific eye and brain conditions, previous thorax surgery and pneumothorax in the past (see online Supplementary Data), the local clinical investigators evaluated the potential risk of spirometry. This included a physical examination, an in depth-medical history and an electrocardiogram. The final decision on the performance of spirometry was made by the local investigators based on all available information. In all study participants, the spirometry measurement was performed within 21 days of TB diagnosis and treatment start.

### Statistical analysis

We displayed the summary statistics of the baseline cohort data, stratified by study site. Continuous variables were summarised using mean, median, range, interquartile range (IQR) and standard deviation (SD). For continuous covariates, statistical testing using the Wilcoxon rank-sum test was done, whereas for categorical covariates statistical testing based on χ^2^ tests were performed. A risk-factor analysis, based on clinically relevant covariates, was performed using logistic regression models to analyse the different outcomes of interest in the study: 1) spirometry done vs. not done; 2) valid vs. invalid spirometry results; and 3) impaired vs. not impaired lung function among valid spirometry results. All analyses have been presented considering type 1 error level (alpha) as 0.05 and with a confidence level of 95%. The statistical analyses were performed using CRAN R v4.1.1.0 (R Computing, Vienna, Austria), and tables were produced using the library arsenal.

### Ethics

The study protocol, informed consent form and other study documents were reviewed and approved by all relevant Ethical Committees at each study site. All participants provided informed consent prior to enrolment in the study.

## RESULTS

### Characteristics of the study cohort

Out of 1,987 subjects screened for study participation in the TB Sequel cohort, 557 were not eligible, mainly due to the absence of a positive *Mycobacterium tuberculosis* test result (refer to online Supplementary Data for details on ineligibility). Overall, the majority of the 1,430 eligible participants had signs of advanced TB disease, with about 68% having a sputum positivity grade of 2+ or 3+; over 70% had moderate or advanced lung pathology on CXR and 47.6% were underweight; 42% of participants tested positive for HIV, 40% of whom were not on antiretroviral treatment and about 20% had a CD4 count of <200 cells/µl (Table 1).

**Table 1:** Baseline characteristics of study participants

	Mozambique (*n* = 417) *n* (%)	Tanzania (*n* = 284) *n* (%)	Gambia (*n* = 370) *n* (%)	South Africa (*n* = 359) *n* (%)	Total (*n* = 1,430) *n* (%)
Male sex	269 (64.5)	169 (59.5)	272 (73.5)	221 (61.6)	931 (65.1)
Age, years, mean ± SD (missing: 3)	35 ± 11	36 ± 10	33 ± 11	38 ± 10	35 ± 11
Age group, years (missing: 3)					
<30	147 (35.4)	79 (27.9)	167 (45.1)	71 (19.8)	464 (32.5)
30–39	136 (32.8)	102 (36.0)	101 (27.3)	147 (40.9)	486 (34.1)
40–49	62 (14.9)	56 (19.8)	50 (13.5)	79 (22.0)	247 (17.3)
50–69	58 (14.0)	33 (11.7)	36 (9.7)	48 (13.4)	175 (12.3)
Tachycardia (missing: 1), >100/bpm	156 (37.4)	168 (59.2)	165 (44.6)	143 (39.9)	632 (44.2)
Sputum smear (missing: 3)					
Negative	63 (15.1)	13 (4.6)	32 (8.6)	66 (18.4)	174 (12.2)
Scanty/1+	93 (22.3)	51 (18.0)	39 (10.5)	100 (27.9)	283 (19.8)
2+/3+	260 (62.4)	218 (76.8)	299 (80.8)	193 (53.8)	970 (67.8)
HIV-positive	191 (45.8)	140 (49.3)	27 (7.3)	248 (69.1)	606 (42.4)
CD4/µl in PLWH (missing: 59), cells/mm3					
<200	67 (45.9)	58 (43.3)	10 (43.5)	150 (41.8)	285 (19.9)
200–500	63 (43.2)	59 (44.0)	10 (43.5)	70 (28.7)	202 (36.9)
≥500	16 (11.0)	17 (12.7)	3 (13.0)	24 (9.8)	60 (11.0)
ART in HIV-positives					
No ART	36 (21.6)	32 (24.6)	2 (28.6)	143 (59.8)	213 (39.2)
On ART	131 (78.4)	98 (75.4)	5 (71.4)	96 (40.2)	330 (60.8)
Unknown	24	10	20	9	63
BMI (missing: 1), kg/m^2^					
Normal	215 (51.6)	145 (51.2)	120 (32.4)	179 (49.9)	659 (46.1)
Underweight (<18.5)	178 (42.7)	126 (44.5)	241 (65.1)	135 (37.6)	680 (47.6)
Overweight (≥25)	24 (5.8)	12 (4.2)	9 (2.4)	45 (12.5)	90 (6.3)
Karnofsky Index (missing: 1), mean ± SD	79.6 ± 4.788	74.401 ± 8.655	80.691 ± 4.314	81.657 ± 6.014	79.370 ± 6.487
Anaemia (missing: 124)^*^					
No anaemia	75 (18.9)	80 (35.2)	85 (26.2)	107 (29.9)	347 (26.6)
Mild	116 (29.2)	74 (32.6)	121 (37.3)	125 (34.9)	436 (33.4)
Moderate	181 (45.6)	63 (27.8)	107 (33.0)	111 (31.0)	462 (35.4)
Severe	25 (6.3)	10 (4.4)	11 (3.4)	15 (4.2)	61 (4.7)
Past TB episodes (missing: 1)	51 (12.3)	25 (8.8)	21 (5.7)	65 (18.1)	162 (11.3)
Number of TB symptoms					
1	87 (21.2)	9 (3.2)	2 (0.5)	47 (13.2)	145 (10.2)
2	113 (27.5)	23 (8.1)	27 (7.3)	136 (38.3)	299 (21.1)
>3	211 (51.3)	252 (88.7)	341 (92.2)	172 (48.5)	976 (68.7)
No symptoms	6	0	0	4	10
Cough	404 (97.6)	284 (100.0)	370 (100.0)	339 (94.4)	1397 (97.9)
Fever	159 (38.4)	214 (75.4)	325 (87.8)	95 (26.5)	793 (55.6)
Weight loss	211 (51.0)	251 (88.4)	360 (97.3)	286 (79.7)	1108 (77.6)
Chest X-ray, with cavitation (missing: 57)	95 (24.2)	75 (27.7)	191 (52.0)	125 (36.4)	486 (35.4)
Ralph score (missing: 43),^†^ median [IQR]	20 [10–48]	40 [15–60]	55 [25–70]	40 [15–70]	32 [15–65]
X-ray score^‡^					
Normal	1 (0.3)	2 (0.7)	2 (0.5)	11 (3.1)	16 (1.2)
Minimally advanced	189 (60.0)	32 (11.8)	36 (9.8)	98 (27.7)	355 (27.2)
Moderately advanced	95 (30.2)	166 (61.3)	181 (49.5)	135 (38.1)	577 (44.2)
Far advanced	30 (9.5)	71 (26.2)	147 (40.2)	110 (31.1)	358 (27.4)
Smoking status (missing: 1)					
Current smoker	32 (7.7)	29 (10.2)	37 (10.0)	112 (31.2)	210 (14.7)
Never smoker	286 (68.8)	207 (72.9)	202 (54.6)	191 (53.2)	886 (62.0)
Past smoker	98 (23.6)	48 (16.9)	131 (35.4)	56 (15.6)	333 (23.3)
Pack-years in smokers (missing: 37), median [IQR]	4.2 [1.7–9.0]	4.0 [1.6–10.9]	6.5 [2.4–13.0]	6.0 [2.5–10.9]	5.4 [2.0–11.5]
Pre-existing CLDs*					
Asthma	20	3	2	11	36
COPD	0	1	0	2	3

* WHO definitions for anaemia: non-anaemia: ≥12 mg/dL (women) or ≥13 mg/dL (men); mild: 11.0–11.9 g/dL (women) and 11.0–12.9 g/dL (men); moderate: 8.0–10.9 g/dL (both sexes); severe: <8.0 g/dL (both sexes); Source: World Health Organization. Haemoglobin concentrations for the diagnosis of anaemia and assessment of severity. Geneva, Switzerland: WHO, 2011.

^†^ Ralph AP, et al. A simple, valid, numerical score for grading chest X-ray severity in adult smear-positive pulmonary tuberculosis. Thorax 2010;65(10):863–869.

^‡^ Diagnostic standard and classification of tuberculosis. National Tuberculosis Association, 1940.

SD = standard deviation; PLWH = people living with HIV; ART = antiretroviral therapy; BMI = body mass index; IQR = interquartile range; CLD = chronic lung disease; COPD = chronic obstructive pulmonary disease.

### Performance of spirometry testing

Of 1,430 eligible participants, spirometry testing was not performed in 353 (24.7%). In the majority of them (288/353, 81.6%), tachycardia was reported as relative exclusion criteria (Figure). In the remaining 65 participants, no reason for exclusion was reported. In 344 participants, spirometry was performed, although tachycardia was present. Compared to tachycardic participants excluded from spirometry, these participants had significantly higher blood pressures, lower respiratory rates, higher Karnofsky Index scores, were less likely to be underweight, overweight or to have lost weight, and more likely to be experiencing their first TB episode (Supplementary Table S3). In the adjusted risk factor analysis, study site, tachycardia, respiratory rate, a low Karnofsky Index, severe anaemia and being overweight were associated with having no spirometry performed (Table 2).

**Figure:**
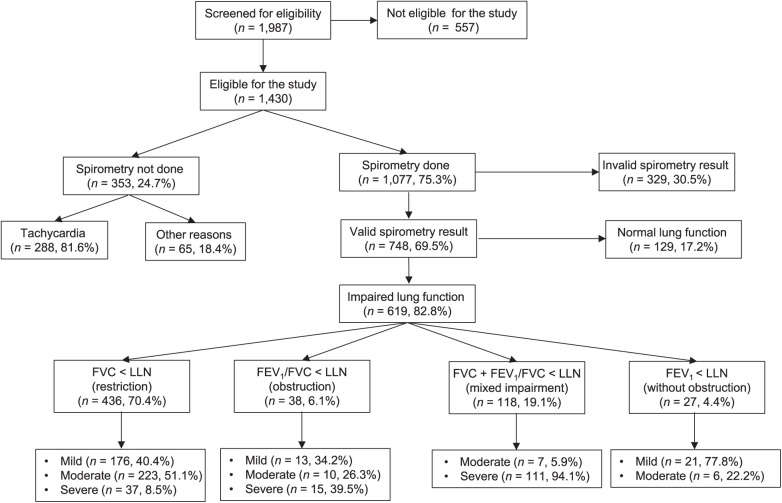
Study flowchart and spirometry outcomes. In total, 557 participants were not eligible for the study. Detailed reasons for non-eligibility are provided in the Supplementary Data. The arrows indicate the hierarchy of boxes and direction of flow. FVC = forced vital capacity; LLN = lower limit of normality; FEV_1_ = forced expiratory volume in 1 sec.

**Table 2:** Risk factors for spirometry ‘not done’ and for ‘invalid’ spirometry results*

Variable	Not done, univariate analysis^†^		Not done, multivariate analysis^‡^		Invalid, univariate analysis^†^		Invalid, multivariate analysis^‡^	
	OR (95% CI)	*P*-value	OR (95% CI)	*P*-value	OR (95% CI)	*P*-value	OR (95% CI)	*P*-value
Study site								
Mozambique	1.00	—	1.00	—	1.00	—	1.00	—
Tanzania	1.14 (0.77–1.67)	0.516	0.88 (0.47–1.63)	0.681	0.73 (0.49–1.07)	0.106	0.70 (0.40–1.19)	0.224
Gambia	1.27 (0.88–1.84)	0.208	1.59 (0.88–2.90)	0.125	0.70 (0.49–1.00)	0.050	0.79 (0.47–1.39)	0.388
South Africa	1.26 (0.86–1.84)	0.232	2.16 (1.26–3.73)	0.005^§^	0.90 (0.64–1.26)	0.536	1.52 (0.95–2.78)	0.085
Sex								
Male	1.00	—	1.00	—	1.00	—	1.00	—
Female	1.34 (1.02–1.77)	0.036^§^	1.18 (0.78–1.78)	0.444	1.24 (0.94–1.63)	0.135	1.12 (0.75–1.66)	0.573
Age, years	1.01 (0.99–1.02)	0.284	1.01 (0.99–1.03)	0.360	0.99 (0.97–1.00)	0.047^§^	0.99 (0.97–1.00)	0.075
HIV status								
Negative	1.00	—	1.00	—	1.00	—	1.00	—
Positive	1.46 (1.08–1.99)	0.016^§^	1.12 (0.74–1.70)	0.590	0.92 (0.68–1.23)	0.557	0.92 (0.63–1.34)	0.661
HIV/CD4, cells/µl								
HIV-negative/ CD4 ≥200	1.00	—	—	—	1.00	—	—	—
HIV CD4 <200	1.33 (0.94–1.87)	0.104	—	—	0.87 (0.60–1.25)	0.450	—	—
HIV and/or ART								
HIV-negative	1.00	—	—		1.00	—	—	—
HIV, no ART	1.09 (0.69–1.71)	0.705	—	—	0.82 (0.53–1.26)	0.374	—	—
HIV plus ART	1.54 (1.08–2.20)	0.018^§^	—	—	0.92 (0.65–1.30)	0.627	—	—
HIV, unknown ART	2.03 (1.09–3.74)	0.024^§^	—	—	1.18 (0.58–2.28)	0.640	—	—
Karnofsky-index	0.94 (0.91–0.96)	<0.001^§^	0.96 (0.93–0.99)	0.015	1.00 (0.97–1.02)	0.834	1.00 (0.97–1.03)	0.820
BMI								
Normal	1.00	—	1.00	—	1.00	—	1.00	—
Underweight	1.55 (1.16–2.07)	0.003^§^	1.44 (0.99–2.10)	0.060	1.27 (0.96–1.68)	0.090	1.35 (0.96–1.89)	0.082
Overweight	2.00 (1.10–3.56)	0.020^§^	2.36 (1.08–5.05)	0.029^§^	1.42 (0.82–2.42)	0.200	0.87 (0.38–1.86)	0.726
Anaemia^¶^								
Normal	1.00	—	1.00	—	1.00	—	1.00	—
Mild	1.09 (0.73–1.64)	0.664	0.97 (0.60–1.57)	0.889	0.82 (0.58–1.16)	0.258	0.76 (0.52–1.12)	0.170
Moderate	1.37 (0.93–2.02)	0.113	1.17 (0.74–1.88)	0.500	0.82 (0.57–1.17)	0.276	0.75 (0.49–1.15)	0.185
Severe	3.59 (1.87–6.94)	<0.001^§^	3.79 (1.72–8.46)	0.001^§^	0.61 (0.25–1.36)	0.249	0.39 (0.11–1.16)	0.116
Tachycardia								
No	1.00	—	1.00	—	1.00	—	1.00	—
Yes	9.41 (7.01–12.80)	<0.001^§^	6.61 (4.46–9.98)	<0.001^§^	1.29 (0.97–1.70)	0.077	0.96 (0.67–1.38)	0.844
Respiratory rate	1.06 (1.03–1.09)	<0.001^§^	1.07 (1.03–1.10)	<0.001^§^	1.02 (0.99–1.06)	0.163	1.04 (1.00–1.08)	0.050
Smoking status								
Current smoker	1.00	—	1.00	—	1.00	—	1.00	—
Non smoker	1.21 (0.79–1.87)	0.393	1.09 (0.65–1.83)	0.747	1.00 (0.68–1.47)	0.982	1.03 (0.65–1.65)	0.896
Past smoker	0.74 (0.45–1.23)	0.243	0.58 (0.32–1.04)	0.068	0.97 (0.63–1.50)	0.889	1.14 (0.69–1.87)	0.617
Smear result								
Negative	1.00	—	1.00	—	1.00	—	1.00	—
Not negative	0.96 (0.67–1.42)	0.843	1.08 (0.64–1.86)	0.789	0.81 (0.55–1.20)	0.285	0.83 (0.52–1.33)	0.423
Ralph score^#^	1.00 (0.99–1.00)	0.411	—	—	1.00 (0.99–1.00)	0.180	—	—
X-ray score**								
Minimal	1.00	—	1.00	—	1.00	—	1.00	—
Moderately advanced	1.07 (0.73–1.56)	0.725	1.44 (0.92–2.27)	0.116	0.91 (0.65–1.30)	0.615	0.98 (0.66–1.45)	0.901
Far advanced	1.00 (0.66–1.52)	0.989	1.12 (0.67–1.88)	0.675	0.80 (0.54–1.20)	0.281	0.96 (0.61–1.52)	0.857
Cavities								
No	1.00	—	—	—	1.00	—	—	—
Yes	0.83 (0.62–1.12)	0.219	—	—	0.97 (0.73–1.29)	0.854	—	—
Past TB								
No	1.00	—	1.00	—	1.00	—	1.00	—
Yes	1.78 (1.17–2.70)	0.007^§^	1.46 (0.85–2.47)	0.164	0.76 (0.48–1.17)	0.224	0.90 (0.52–1.33)	0.701

* The first regression analysis analyses risk factors for spirometry done vs. spirometry not done. In the next step among the individuals where spirometry was done, we analyse risk factors for valid spirometry vs. invalid spirometry.

^†^ Adjusted for site and tachycardia.

^‡^ Mutually adjusted for all variables in the model.

^§^ Statistically significant.

^¶^ WHO definitions for anaemia: non-anaemia: ≥12 mg/dL (women) or ≥13 mg/dL (men); mild: 11.0–11.9 g/dL (women) and 11.0–12.9 g/dL (men); moderate: 8.0–10.9 g/dL (both sexes); severe: <8.0 g/dL (both sexes); Source: World Health Organization. Haemoglobin concentrations for the diagnosis of anaemia and assessment of severity. Geneva, Switzerland: WHO, 2011.

^#^ Ralph AP, et al. A simple, valid, numerical score for grading chest x-ray severity in adult smear-positive pulmonary tuberculosis. Thorax 2010;65(10):863–869.

^**^ Diagnostic standard and classification of tuberculosis. National Tuberculosis Association, 1940.

OR = odds ratio; CI = confidence interval; ART = antiretroviral therapy; BMI = body mass index.

Of 1,077 participants in whom spirometry was performed, 329 (30.5%) had an invalid spirometry result (Figure). Furthermore, 14.8% (111/748) of valid spirometry results were graded as ‘E’, that is, only one acceptable curve was produced by the respective participant and, thus, reproducibility of the spirometry result could not be demonstrated. In the adjusted risk factor analysis, we could not identify any factor that was associated with an invalid spirometry result (Table 2). Moreover, investigators reported artefacts due to cough, early limitation of exhalation time and insufficient effort as the main reasons for invalid spirometry tests. To note, although symptoms such as tiredness, dizziness or physical exhaustion led to stopping the spirometry procedure in several participants without achieving a valid test result, no adverse events requiring medical intervention were reported in relation to spirometry. Of note, among the 344 participants, in whom spirometry was performed despite having tachycardia, a similar proportion of invalid results (33.7%, 116/344) compared to non-tachycardic participants (28.9%, 212/712), and no specific adverse events, were reported.

### Spirometry outcomes

Of 748 participants with a valid spirometry result, 619 (82.8%) had impaired lung function, with the majority (70.6%, 436/619) having a FVC value below the LLN (Figure). Only 6.1% (38/619) of participants with lung impairment had an isolated reduced FEV_1_/FVC ratio and 19% (118/619) had mixed impairment, i.e., an FEV_1_/FVC ratio below LLN with an FVC below LLN (Figure). However, the proportion of participants with severe lung impairment was greater among those with reduced FEV_1_/FVC ratio (obstruction and mixed impairment, 39.5% and 94.1%, respectively) compared to participants with low FVC (8.5%), resulting in overall 26.3% (163/619) with severe lung impairment among those with abnormal spirometry (Figure). Independent risk factors associated with severe lung impairment in this subgroup (Table 3) were being underweight, advanced pathology on CXR, tachycardia and a higher respiratory rate. Participants at the South African study site and those of older age were significantly less likely to have severe lung impairment on spirometry testing (Table 3).

**Table 3:** Risk factors for moderate or severe lung impairment in spirometry

Variable		Lung impairment, univariate analysis*			Lung impairment, multivariate analysis^†^		
		OR (95% CI)		*P*-value	OR (95% CI)		*P*-value
Study site							
	Mozambique	1.00	—	—	1.00	—	—
	Tanzania	0.90	0.54–1.48	0.663	0.62	0.31–1.24	0.179
	Gambia	1.95	1.16–3.35	0.013^‡^	0.75	0.38–1.51	0.422
	South Africa	0.31	0.20–0.48	<0.001^‡^	0.29	0.15–0.55	<0.001^‡^
Sex							
	Male	1.00	—	—	1.00	—	—
	Female	0.92	0.64–1.34	0.664	0.95	0.56–1.62	0.85
Age		0.97	0.95–0.98	<0.001^‡^	0.96	0.95–0.98	<0.001^‡^
HIV status							
	Negative	1.00	—	—			
	Positive	0.68	0.47–0.99	0.046^‡^			
HIV/CD4, cells/µl							
	HIV-negative	1.00	—	—	1.00	—	—
	HIV <200	0.65	0.43–1.01	0.051	0.83	0.47–1.47	0.517
HIV/ART							
	HIV-negative	1.00	—	—			
	HIV, no ART	0.70	0.42–1.16	0.159			
	HIV plus ART	0.69	0.45–1.08	0.106			
Karnofsky Index		0.99	0.96–1.02	0.465	1.04	1.00–1.08	0.051
BMI							
	Normal	1.00	—	—	1.00	—	—
	Underweight	3.55	2.36–5.44	<0.001^‡^	2.91	1.83–4.71	<0.001^‡^
	Overweight	0.53	0.27–1.03	0.063	0.51	0.21–1.24	0.140
Anaemia^§^							
	Normal	1.00	—	—	1.00	—	—
	Mild	1.38	0.89–2.13	0.146	0.94	0.57–1.55	0.812
	Moderate/severe	1.98	1.26–3.12	0.003^‡^	1.53	0.87–2.70	0.137
Tachycardia							
	No	1.00	—	—	1.00	—	—
	Yes	2.76	1.82–4.27	<0.001^‡^	1.79	1.06–3.06	0.031^‡^
Respiratory rate		1.107	1.05–1.17	<0.001^‡^	1.10	1.03–1.17	0.004^‡^
Smoking status							
	Current smoker	1.00	—	—	1.00	—	—
	Non smoker	1.12	0.70–1.79	0.633	1.66	0.92–3.00	0.089
	Past smoker	1.15	0.66–2.00	0.619	1.49	0.78–2.83	0.224
Smear result							
	Negative	—	—	—	—	—	—
	Not negative	1.97	1.19–3.23	0.008^‡^	1.15	0.63–2.08	0.654
Cavities							
	No	1.00	—	—			
	Yes	1.232	0.85–1.81	0.278			
X-ray score^¶^							
	Minimal advanced	1.00	—	—	1.00	—	—
	Moderately advanced	1.54	1.00–2.39	0.052^‡^	1.48	0.83–2.67	0.186
	Far advanced	4.37	2.53–7.73	<0.001^‡^	3.53	1.44–8.84	0.006^‡^
Ralph Score^#^		1.02	1.01–1.03	<0.001^‡^			
Past TB							
	No	1.00	—	—	1.00	—	—
	Yes	1.18	0.70–2.05	0.547	1.00	0.99–1.02	0.468

* Adjusted for site.

^†^ Mutually adjusted for all variables in the model.

^‡^ Statistically significant.

^§^ WHO definitions for anaemia: non-anaemia: ≥12 mg/dL (women) or ≥13 mg/dL (men); mild: 11.0–11.9 g/dL (women) and 11.0–12.9 g/dL (men); moderate: 8.0–10.9 g/dL (both sexes); severe: <8.0 g/dL (both sexes); Source: World Health Organization. Haemoglobin concentrations for the diagnosis of anaemia and assessment of severity. Geneva, Switzerland: WHO, 2011.

^¶^ Diagnostic standard and classification of tuberculosis. National Tuberculosis Association, 1940.

^#^Ralph AP, et al. A simple, valid, numerical score for grading chest x-ray severity in adult smear-positive pulmonary tuberculosis. Thorax 2010;65(10):863–869.

OR = odds ratio; CI = confidence interval; ART = antiretroviral therapy; BMI = body mass index.

## DISCUSSION

To our knowledge, the TB Sequel cohort is currently the largest prospective study of PTLD worldwide to explore the characteristics and evolution of lung function impairment from TB diagnosis into the post-treatment period. In this study, of the spirometry assessment performed at TB diagnosis we found that spirometry was safe in acutely diseased TB participants with no reported clinical adverse events. However, a high proportion of tests were not performed or were discarded as invalid, and re-education and training of staff was important. In those with reportable spirometry at TB diagnosis, more than 80% had abnormal lung function, with 26% having severe lung impairment.

Unlike many previous clinical PTLD studies, we performed spirometry within 21 days after TB diagnosis. We implemented spirometry testing at four different African TB research sites where spirometry was previously unavailable. The findings reported can be viewed as a ‘proof of concept’ strategy on the feasibility of introducing spirometry testing into clinical management of TB, e.g., in NTPs. It should be noted, however, that a high proportion of tests were not performed or were discarded as invalid; 80% of participants excluded from spirometry assessment had tachycardia, which was a relative contraindication per protocol. Of note, 388 tachycardic participants who underwent spirometry showed the same performance in spirometry as non-tachycardic participants. This calls into question the labelling of tachycardia in isolation as a contraindication. It must be acknowledged, however, that tachycardic participants who were excluded from spirometry may have been in such poor clinical condition that the investigators considered spirometry generally unfeasible, with tachycardia being the only symptom that could be reported as a relative contraindication. Supporting this notion, our data demonstrate that investigators excluded participants from testing with advanced TB disease and comorbidities, which may be perceived by personnel as a barrier to the performance of reliable and interpretable spirometry. We also observed that participants at CHRU (South Africa) were more likely to be excluded from spirometry, a fact, which may partially explain the better lung function results for the South African participants who underwent spirometry testing.

We identified two main sources of invalid results: 1) artefacts in the curves resulting unsurprisingly from cough, or insufficient familiarity, understanding or ability of the participants to perform spirometry; 2) interrupted testing due to exhaustion or dizziness in the participants. These findings highlight the importance of quality control, audit and corrective measures when implementing spirometry in a new setting. During the course of the study, we addressed both the ‘exclusion’ of participants and proportion of ‘invalid’ spirometry results by site-specific, online and face-to-face re-training. Study personnel were encouraged to test all participants who were clinically stable and re-trained in the education and motivation of participants during spirometry, which led to an improvement in the proportion of participants with valid and reproducible spirometry results during our study follow-up. A decrease in the proportion of TB patients who did not meet the reproducibility criteria in the spirometry assessment during TB treatment was also shown in a trial evaluating adjunctive host-directed therapies for pulmonary TB.^14^

In those 50% of study participants with a valid spirometry result, more than 80% had abnormal lung function, with about 50% of them having moderate or severe lung impairment. The high proportion of participants with a FVC below the LLN, as well as the lower risk for lung impairment in participants from South Africa, may be partly explained by an inappropriateness of the GLI reference equations, resulting in misclassification of FVC impairment in participants.^15^ In contrast, the FEV_1_/FVC ratio is not as dependent on reference equations. In addition, body and muscle weakness in critically ill patients may have resulted in the measurement of falsely low FVC values rather than falsely low FEV_1_ values, leading to a bias toward a normal or high FEV_1_/FVC ratio and underreporting of obstruction at the start of TB treatment. This is why we did not assess risk factors separately for obstructive and restrictive lung impairment in this study.

The results of the risk factor analysis indicate that early TB diagnosis is key to mitigating TB-related morbidity burden. Both low body mass index and severe pathology on CXR, both indicators of advanced TB disease, were associated with lung impairment. Because the study team tended to exclude sicker participants from spirometry, it is likely that the 80% lung impairment at the time of TB diagnosis represents an underestimation of the true burden. To note, one European study showed that lung impairment at TB diagnosis was associated with adverse TB outcomes such as treatment failure and death during treatment.^16^ The value of lung impairment in spirometry at TB treatment initiation for the prediction of future PTLD and long-term morbidity is currently unknown. Few, smaller, prospective clinical studies have shown that while lung function improves both during and after TB treatment, it does not return to standard normal values in a significant proportion of TB survivors.^17^–^20^

Abnormal FEV_1_ has been associated with increased morbidity and mortality in epidemiological studies of both the general population and populations with COPD.^21^,^22^ It is unknown whether this is also the case for patients with PTLD. However, given the high number of TB survivors globally^23^ and in sub-Saharan Africa specifically, there is great cause for concern that a large proportion of TB patients may be at risk of prolonged lung impairment, which may be also a relevant cause of increased morbidity after TB treatment completion.

In conclusion, we found that performing spirometry in newly diagnosed TB patients, although challenging, is both safe and feasible in low- and middle-income settings. Including patients with isolated tachycardia, may increase the available spirometry results by up to 20%, and was found to be safe in our cohort. Our experience shows that spirometry, when performed at TB diagnosis may remain unsuccessful in up to 30% of patients, especially in patients with advanced clinical disease and prominent symptoms. Despite these limitations, the high proportion of TB patients with abnormal spirometry at diagnosis underscores the importance of performing spirometry during TB treatment, and the need for prospective clinical studies to better understanding the long-term implications of these abnormalities for TB treatment outcomes and the development of subsequent PTLD.
