# Supplementary material for: Performance of spirometry assessment at TB diagnosis
Source: Int J Tuberc Lung Dis. 2023 Nov 1;27(11):850–7. doi: 10.5588/ijtld.23.0040 (PMC10599411; doi:10.5588/ijtld.23.0040)
Supplement: Supplementary file 1 [file iutld_ijtld_23.0040_supplementarydata1.pdf]

http://dx.doi.org/10.5588/ijtld.23.0040-

SUPPLEMENTARY DATA

Performance of spirometry assessment at TB diagnosis

A. Performance of spirometry and related quality control procedures:

All study procedures (within 21 days after TB treatment start) related to spirometry testing, quality control of spirometry results and data analysis were performed according to ATS/ERS guidelines published in 2005 (1), using the ndd EasyOne® or Easy on-PC device (NDD Medizintechnik AG, Technoparstrasse 1, CH-8005 Zürich, Schweiz). Spirometry measurements within 21 days after TB diagnosis were performed without application of bronchodilators. For this study, a list of potential medical contraindications, which could be adversely affected by the maximal pressures in thorax and abdomen as well as by the increase in myocardial demand that are associated with spirometry was generated by the involved investigators, external monitors and experts, as such a list was not included in ARS/ERS guidelines from 2005 (1, 2). The following conditions were considered **relative contraindications for spirometry**, which requested further discussion among the site principle investigator and local investigators in order to decide, whether or not spirometry can be performed in a specific participant: **tachycardia >100 beats/minute, blood pressure > 180/110 mmHG, chest/abdominal surgery in last 3 months, cardiac infarction in last 3 months, hospitalization due to cardiac decompensation in last 1 month, unstable angina pectoris, detached retina/eye surgery in last 3 months, 3rd trimester pregnancy, haemoptysis of unknown origin or TB related in last 1 month, pneumothorax in last 3 months, other acute illnesses**. Disposable spirettes were used and changed for each participant. Investigations were either performed in a private area outdoors (NIMR-MMRC, Tanzania and INS, Mozambique) or in well ventilated rooms with more than one opened window (MRC, The Gambia and CHRU, South Africa), which were cleaned and not entered for 30 minutes after each spirometry investigation. Study staff was wearing N95 or FFP2 masks during the investigation and in the whole area, where spirometry assessments were performed. The spirometric manoeuvre was performed in a sitting position. Nose clips were not used by most sites, instead the participants were shown how to pinch their nose during testing.

Spirometry data were stored electronically using EasyWare software (NDD Medizintechnik AG, Technoparstrasse 1, CH-8005 Zürich, Schweiz) and uploaded onto a server hosted by the study team at Ludwig- Maximilian-University in Munich (LMU Klinikum). Internal and external quality control (2 reviewers in total) was performed including 100% of produced spirometry curves. Internal quality control was performed chronological within 1 week after spirometry measurement, external quality control was performed in batches of 50 curves immediately after study start, and then in batches of

100-200 curves from 2018 onwards. Errors of each spirometry attempt were categorized as shown in **supplement table 1** below. Afterwards, each spirometry attempt was quality graded according to **supplementary table 2**. After the release of an updated version of ATS/ERS spirometry guidelines in 2019 (3), all spirometry attempts with a grading of “2” or below (**supplementary table 2**) were individually reviewed again by an expert from PATS (Pan African Thoracic Society) and graded according to the new guidelines (2). For this manuscript, all spirometry results of at least grade “E” were considered as **valid** (**supplement figures 1, 2**), (3). In total, 14.8% (111 out of 748) of spirometry results included in the final analysis were graded with “E”, which is allowing for one acceptable curve only or repeatability of FEV1 and FVC within >250ml (2).

Supplementary Table S1: List of potential errors of spirometry curves (used in TB Sequel study until end of January 2020)

Error	Abbreviation	Definition	Reference/ source
High PEFT	p (PEFT)	PEFT ≥150msecs	BOLD QC requirements, which relaxed the ndd cut off of 120ms
High BEV	b (BEV)	BEV ≥150ml<br>**AND**<br>BEV ≥5% of FVC	ATS criteria, NIOSH, BOLD QC requirements state that for a curve to be included BEV must be <5% or <150ml, whichever is greater.
Non-maximal effort	e (effort)	Marked lack of peak, indicating weak blast<br>**OR**<br>Markedly reduced peak compared to other curves, indicating poor filling of lungs at start of test	BOLD QC requirements<br>ATS criteria<br>NIOSH guidelines
Early termination of expiration	t (termination)	Insufficient expiratory phase on volume-time curve – duration of expiration for < 6 secs OR failure to reach plateau of ≥1 sec<br>**OR**<br>Sharp early drop to 0 on flow-volume curve	BOLD QC requirements<br>ATS criteria<br>NIOSH guidelines
Extra breath	x (extra)	Visible extra breath on flow-volume and or the volume-time curves	BOLD QC requirements<br>NIOSH guidelines
Glottis closure that influences measurement	g (glottis)	Abrupt flat line on volume-time curve, with sharp drop to 0 on flow-volume curve	BOLD QC requirements<br>ATS criteria<br>NIOSH guidelines
Leak	l (leak)	Descent of volume-time curve, after peak is reached, with ‘back-track’ of flow-volume curve at the end of expiration	BOLD QC requirements<br>ATS criteria<br>NIOSH guidelines
Obstructed mouthpiece	o (obstruction)	Artefact in the flow-volume and volume-time curves, felt to be significant enough to affect measurement	BOLD QC requirements<br>ATS criteria<br>NIOSH guidelines

Cough that affects measurement	c (cough)	Cough within 1st second which is likely to alter FEV1, or a later cough which causes early termination.	BOLD QC requirements<br>ATS criteria<br>NIOSH guidelines
Zero flow error	z (zero)	Continuous rise of volume-time curve, with no plateau, and long tail on flow-volume curve, which is felt related to error rather than obstructive impairment	BOLD QC requirements<br>NIOSH guidelines

Supplementary Table S2: Quality grading of usable curves, including repeatability assessment (used in TB Sequel study until end of January 2020)

Validity classification for FEV1 and FVC	Abbreviatio n	Definition if FEV or FV volume ≥1 L	Difference in definition, if FEV or FVC volume <1L
Unable to assess	0	< 2 usable curves, so unable to compare	No change
Not valid	1	≥2 usable curves<br>**AND**<br>Difference between highest usable and second highest usable FEV_1 >250ml<br><br>Difference between highest usable FVC and second highest usable FVC >250ml	Difference is >200ml
Borderline	2	≥2 usable curves<br>**AND**<br>Difference between highest usable FEV_1 and second highest usable FEV_1 is >200ml but ≤250ml<br><br>Difference between highest usable FVC and second highest usable FVC is >200ml but ≤250ml	Difference is >150ml but ≤200ml
**Valid** – BOLD QC criteria	**3**	≥2 usable curves<br>**AND**<br>Difference between highest usable FEV_1 and second highest usable FEV_1 is >150ml but ≤200ml<br><br>Difference between highest usable FVC and second highest usable FVC >150ml but ≤200ml	Difference is >100ml but ≤150ml
**Valid** – ATS QC criteria	**4**	≥2 usable curves<br>**AND**<br>Difference between highest usable FEV_1 and second highest usable FEV_1 ≤150ml	Difference is ≤150ml

		Difference between highest usable FVC and second highest usable FVC ≤150ml	

Supplementary Figure S1: Summary of Acceptability, Usability, and Repeatability Criteria for FEV1 and FVC according to new ATS/ERS guidelines (used in TB Sequel study from February 2020 onwards)

Acceptability and Usability Criterion	Required for Acceptability		Required for Usability	
	FEV_1	FVC	FEV_1	FVC
Must have BEV ≤5% of FVC or 0.100 L, whichever is greater	Yes	Yes	Yes	Yes
Must have no evidence of a faulty zero-flow setting	Yes	Yes	Yes	Yes
Must have no cough in the first second of expiration*	Yes	No	Yes	No
Must have no glottic closure in the first second of expiration*	Yes	Yes	Yes	Yes
Must have no glottic closure after 1 s of expiration	No	Yes	No	No
Must achieve one of these three EOFE indicators: 1. Expiratory plateau (≤0.025 L in the last 1 s of expiration) 2. Expiratory time ≥15 s 3. FVC is within the repeatability tolerance of or is greater than the largest prior observed FVC[†]	No	Yes	No	No
Must have no evidence of obstructed mouthpiece or spirometer	Yes	Yes	No	No
Must have no evidence of a leak	Yes	Yes	No	No
If the maximal inspiration after EOFE is greater than FVC, then FIVC − FVC must be ≤0.100 L or 5% of FVC, whichever is greater[‡]	Yes	Yes	No	No

Repeatability criteria (applied to acceptable FVC and FEV_1 values)

Age >6 yr: The difference between the two largest FVC values must be ≤0.150 L, and the difference between the two largest FEV_1 values must be ≤0.150 L

Age ≤6 yr: The difference between the two largest FVC values must be ≤0.100 L or 10% of the highest value, whichever is greater, and the difference between the two largest FEV_1 values must be ≤0.100 L or 10% of the highest value, whichever is greater

Definition of abbreviations: BEV = back-extrapolated volume; EOFE = end of forced expiration; $FEV_{0.75}$ = forced expiratory volume in the first 0.75 seconds; FIVC = forced inspiratory VC.

The grading system (Table 10) will inform the interpreter if values are reported from usable maneuvers not meeting all acceptability criteria.

*For children aged 6 years or younger, must have at least 0.75 seconds of expiration without glottic closure or cough for acceptable or usable measurement of $FEV_{0.75}$.

[†]Occurs when the patient cannot expire long enough to achieve a plateau (e.g., children with high elastic recoil or patients with restrictive lung disease) or when the patient inspires or comes off the mouthpiece before a plateau. For within-maneuver acceptability, the FVC must be greater than or within the repeatability tolerance of the largest FVC observed before this maneuver within the current prebronchodilator or the current post-bronchodilator testing set.

[‡]Although the performance of a maximal forced inspiration is strongly recommended, its absence does not preclude a maneuver from being judged acceptable, unless extrathoracic obstruction is specifically being investigated.

Legend to supplement figure 1: this figure is identical to Table 7 in Standardization of Spirometry 2019 Update by ATS/ERS (3).

Supplementary Figure S2: Grading System for FEV1 and FVC according to new ATS/ERS guidelines (used in TB Sequel study from February 2020 onwards)

Grade	Number of Measurements	Repeatability: Age >6 yr	Repeatability: Age ≤6 yr*
A	≥3 acceptable	Within 0.150 L	Within 0.100 L*
B	2 acceptable	Within 0.150 L	Within 0.100 L*
C	≥2 acceptable	Within 0.200 L	Within 0.150 L*
D	≥2 acceptable	Within 0.250 L	Within 0.200 L*
E	≥2 acceptable	>0.250 L	>0.200 L*
	OR 1 acceptable	N/A	N/A
U	0 acceptable AND ≥1 usable	N/A	N/A
F	0 acceptable and 0 usable	N/A	N/A

Definition of abbreviation: N/A = not applicable.

The repeatability grade is determined for the set of prebronchodilator maneuvers and the set of post-bronchodilator maneuvers separately. The repeatability criteria are applied to the differences between the two largest FVC values and the two largest FEV_1 values. Grade U indicates that only usable but not acceptable measurements were obtained. *Although some maneuvers may be acceptable or usable at grading levels lower than A, the overriding goal of the operator must be to always achieve the best possible testing quality for each patient.* Adapted from Reference 114.

*Or 10% of the highest value, whichever is greater; applies for age 6 years or younger only.

Legend to Supplementary Figure S2: this figure is identical to Table 10 in Standardization of Spirometry 2019 Update by ATS/ERS (3).

B. <u>References, prediction equations, and severity grading for statistical analysis:</u>

For the analysis of spirometry results we used the prediction equations for "others" standardized for age, sex and height, which were published by the Global Lung Initiative (GLI), (4). Based on the GLI standard for "others" we calculated the estimate for the predicted values for FEV1, FVC and the ratio of FEV1: FVC for healthy individuals having the same age, sex and height. Based on GLI standard for "others" we also obtained the lower limit of normality (LLN) for each of the predicted values for FEV1, FVC and FEV1/FVC-ratio. Further, this allowed us to compute the z-scores for observed values for FEV1, FVC and FEV1/FVC-ratio. Lung impairment (LI) was defined if either of the observed values for FEV1 or FVC or the ratio of FEV1: FVC was lower than the LLN to the predicted value. Participants with LI were categorized in 4 subgroups (restriction, obstruction, mixed and FEV1 below the LLN but no obstruction) and further categorized according to the severity of LI as follows.

1. Restriction: FVC observed (obs) less than LLN FVC predicted (and FEV1:FVC-ratio ≥LLN), we graded it as
    - Mild when FVC obs >= 0.85 * LLN FVC
    - Moderate when 0.55 * LLN FVC <= FVC obs < 0.85 * LLN FVC
    - Severe when FVC obs < 0.55 * LLN FVC
2. Obstruction, FEV1: FVC-ratio observed (obs) less than LLN FEV1: FVC-ratio predicted, we graded it as
    - Mild when z score FEV1 obs >= -2
    - Moderate when -2.5 <= z score FEV1 obs. < -2
    - Severe when z score FEV1 obs < -2.5
3. Mixed, then ratio FEV1: FVC observed (obs) less than LLN ratio FEV1: FVC predicted and FVC observed less than LLN FVC predicted, we graded it according to the z-score for FEV1 or FVC, depending on which was lowest z-score of FVC obs or z-score of FEV1/FVC-ratio obs. As in all participants with mixed lung impairment the z-score for FEV1/FVC-ratio obs was lower than the z-score for FVC obs, we graded as following:
    - Mild when z score FEV1 obs >= -2
    - Moderate when -2.5 <= z score FEV1 obs. < -2
    - Severe when z score FEV1 obs < -2.5
4. When FEV1 observed (obs) less than LLN FEV1 predicted only, but FVC obs and ratio FEV1: FVC obs were not impaired, then we graded it by
    - Mild when z score FEV1 obs >= -2
    - Moderate when -2.5 <= z score FEV1 obs < -2

C. Eligibility assessment for study participation:

The number of participants, who did not meet all **inclusion criteria** was 509. One or more of the following inclusion criteria could be not fulfilled among screened participants. The list below provides the information in how many participants the respective inclusion criteria was not fulfilled.

- Available microbiological evidence for TB infection (positive sputum-based MTB/RIF test or positive sputum culture): 451*
- Agreement to follow-up for 24 months: 62
- Agreed with collection and storage of all blood, sputum and urine samples: 18
- Resident in study area: 13
- Willing to start anti-tuberculosis treatment: 10
- Willing to be tested for HIV: 8
- Signed informed consent form: 5
- Older than 18 years: 1

*The study site at MRC in Gambia acts as a service provider for TB diagnosis in the local communities. The site staff consented all symptomatic subjects prior TB testing for participation in TB Sequel. This resulted in a high number of 67.6% (305/451) uneligible participants at MRC due to a negative MTB screening result.

The number of participants, in whom all **exclusion criteria** were not absent was 48. One or more of the following exclusion criteria could be present among screened participants. The list below provides the information in how many participants the respective exclusion criteria was present.

- Participant is incapable of producing a sputum sample: 24
- Participant has severe medical or psychiatric condition, which could have influence on the adherence and outcome of TB treatment: 15
- Participant is on TB treatment for longer than 7 days at the time point of eligibility assessment for this study: 9
- Participant is imprisioned: 4
- Participant takes part in an investigational TB drug trial: 2

D. Results

Supplementary Table S3: Comparison of characteristics in tachycardic patients, in whom spirometry was done versus not done at TB diagnosis

	Done (N=344)	Not done (N=288)	Total (N=632)	p value
**SiteID**				0.608[1]
INS Mzb	89 (25.9%)	67 (23.3%)	156 (24.7%)	
MMRC Tz	91 (26.5%)	77 (26.7%)	168 (26.6%)	
MRC Gmb	83 (24.1%)	82 (28.5%)	165 (26.1%)	
WITS Rsa	81 (23.5%)	62 (21.5%)	143 (22.6%)	
**Sex**				0.065[1]
Female	132 (38.4%)	132 (45.8%)	264 (41.8%)	
**Age**				0.511[2]
Mean (SD)	34 (10)	34 (11)	34 (10)	
**Syst. blood pressure**				**0.013[2]**
Mean (SD)	109 (15)	106 (16)	108 (16)	
**Diast. blood pressure**				**0.035[2]**
Mean (SD)	73 (12)	71 (11)	73 (12)	
**Respiratory rate**				**< 0.001[2]**
Mean (SD)	23 (6)	25 (6)	244 (6)	
**HIV Status**				0.169[1]
Positive	150 (43.6%)	142 (49.3%)	292 (46.2%)	
**BMI categories**				**0.015[1]**
Normal	149 (43.3%)	96 (33.4%)	245 (38.8%)	
Underweight	185 (53.8%)	174 (60.6%)	359 (56.9%)	
Overweight	10 (2.9%)	17 (5.9%)	27 (4.3%)	
**Karnofsky score**				**0.005[2]**
Mean (SD)	79 (6)	77 (8)	78 (7)	
Min - Max	60- 90	40- 90	40- 90	
**Anaemia**				0.270[1]
No Anaemia	65 (21.0%)	48 (19.1%)	113 (20.2%)	
Mild	96 (31.1%)	71 (28.3%)	167 (29.8%)	
Moderate	134 (43.4%)	111 (44.2%)	245 (43.8%)	
Severe	14 (4.5%)	21 (8.4%)	35 (6.2%)	
**Past TB episodes**				**< 0.001[1]**
Yes	18 (5.2%)	43 (14.9%)	61 (9.7%)	
**Cough**				0.732[1]
Yes	340 (98.8%)	282 (98.3%)	622 (98.6%)	
**Weight loss**				**0.022[1]**
Yes	277 (80.5%)	251 (87.5%)	528 (83.7%)	

Legend to supplement table 3:

1. Pearson's Chi-squared test with simulated p-value (based on 10000 replicates)
2. Kruskal-Wallis rank sum test

E. List of abbreviations:

ATS	American Thoracic Society
BOLD	Burden of Lung Disease- study
ERS	European Respiratory Society
GLI	Global Lung Initiative
FEV1	Forced Expiratory Volume in 1 second
FVC	Forced Vital Capacity
LI	Lung Impairment
LLN	Lower Limit of Normality
MTB	Mycobacterium tuberculosis
ndd	NDD Medical Technologies
NIOSH	National Institute for Occupational Safety and Health
obs	observed
PATS	Pan African Thoracic Society
QC	Quality Control
TB	Tuberculosis

F. References:

1. Miller MR, Hankinson J, Brusasco V, Burgos F, Casaburi R, Coates A, et al. Standardisation of spirometry. Eur Respir J. 2005;26(2):319-38.

2. Miller MR, Crapo R, Hankinson J, Brusasco V, Burgos F, Casaburi R, et al. General considerations for lung function testing. Eur Respir J. 2005;26(1):153-61.

3. Graham BL, Steenbruggen I, Miller MR, Barjaktarevic IZ, Cooper BG, Hall GL, et al. Standardization of Spirometry 2019 Update. An Official American Thoracic Society and European Respiratory Society Technical Statement. Am J Respir Crit Care Med. 2019;200(8):e70-e88.

4. Quanjer PH, Stanojevic S, Cole TJ, Baur X, Hall GL, Culver BH, et al. Multi-ethnic reference values for spirometry for the 3-95-yr age range: the global lung function 2012 equations. Eur Respir J. 2012;40(6):1324-43.
